# Targeted multi-epitope switching enables straightforward positive/negative selection of CAR T cells

**DOI:** 10.1038/s41434-021-00220-6

**Published:** 2021-02-01

**Authors:** Laura Mosti, Lukas M. Langner, Kay O. Chmielewski, Patrick Arbuthnot, Jamal Alzubi, Toni Cathomen

**Affiliations:** 1 Institute for Transfusion Medicine and Gene Therapy, Medical Center—University of Freiburg, Freiburg, Germany; 2 Center for Chronic Immunodeficiency (CCI), Medical Center—University of Freiburg, Freiburg, Germany; 3 Ph.D. Program, Faculty of Biology, University of Freiburg, Freiburg, Germany; 4 M.D. Program, Faculty of Medicine, University of Freiburg, Freiburg, Germany; 5 Wits/SAMRC Antiviral Gene Therapy Research Unit, School of Pathology, Faculty of Health Sciences, University of the Witwatersrand, Johannesburg, South Africa; 6 Faculty of Medicine, University of Freiburg, Freiburg, Germany

**Keywords:** Immunotherapy, Gene therapy

## Abstract

Chimeric antigen receptor (CAR) T cell technology has enabled successfully novel concepts to treat cancer patients, with substantial remission rates in lymphoid malignancies. This cell therapy is based on autologous T lymphocytes that are genetically modified to express a CAR that recognizes tumor-associated antigens and mediates the elimination of the respective tumor cells. Current limitations include laborious manufacturing procedures as well as severe immunological side effects upon administration of CAR T cells. To address these limitations, we integrated RQR8, a multi-epitope molecule harboring a CD34 epitope and two CD20 mimotopes, alongside a CD19-targeting CAR, into the *CD52* locus. Using CRISPR-Cas9 and adeno-associated virus-based donor vectors, some 60% of genome-edited T cells were CAR^+^/CD20^+^/CD34^+^/CD52^−^ without further selection. This could be increased to >95% purity after CD34 tag-based positive selection. These epitope-switched CAR T cells retained cell killing competence against CD19^+^ tumor cells, and were resistant to alemtuzumab (anti-CD52) but sensitive to rituximab (anti-CD20) in complement-dependent cytotoxicity assays. In conclusion, gene editing-based multiple epitope switching represents a promising development with the potential to improve both the manufacturing procedure as well as the clinical safety of CAR T cells.

## Introduction

**Fig. 1:**
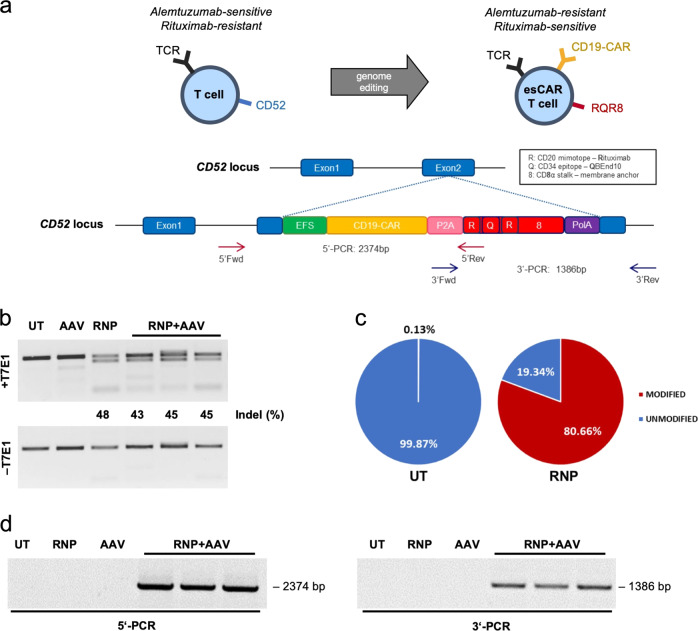
Genome editing in primary T cells — genotypic analysis. **a** Schematic of gene targeting strategy. Integration of a CAR–RQR8 construct is targeted to exon 2 of the *CD52* locus. Gene targeting by homology-direct repair is mediated by a CRISPR-Cas9 nuclease in combination with an AAV6 donor vector that harbors the CAR–RQR8 expression cassette flanked by ≈500 bp long homology arms. EFS, elongation factor 1α short promoter; CD19-CAR, CD19-targeting CAR; P2A, 2A self-cleaving peptide of porcine teschovirus; RQR8, see schematic; polyA, poly-adenylation signal. **b** Genotyping by T7E1 assay. The percentage of cleaved products is indicated. **c** Genotyping by NGS analysis. Pie charts show fractions of alleles with indels. **d** Genotyping by in–out junction PCR. Primer binding sites for 5′ and 3′-junction PCRs as well as expected product lengths are indicated in panel (**a**). Indel, insertion/deletion mutation; UT, untreated T cells; AAV, AAV-transduced T cells; RNP, RNP-electroporated T cells; RNP + AAV, electroporated with RNP and transduced with 1 × 10^4^, 3 × 10^4^, and 5 × 10^4^ genome copies/cell from left to right, respectively.

In the last few years, CD19-targeting chimeric antigen receptor (CAR) T cell therapy showed high rates of complete remission in B cell acute lymphoblastic leukemia (B-ALL), and moderate response rates in both diffuse large B-cell lymphoma (DLBCL) and chronic lymphocytic leukemia [1–3]. Followed by these results, the FDA and the EMA approved new drugs, such as tisagenlecleucel [4] and axicabtagene ciloleucel [5], for the treatment of both relapsed or refractory CD19^+^ B-ALL and DLBCL, respectively [6]. Several research groups are developing yet other CAR T cells to diversify the range of targetable tumor types, including solid tumor entities [7]. Although CD19-CAR T cell therapy has already proven to successfully reduce tumor burden in cancer patients, further engineering is needed to enhance safety, specificity, and efficacy of this novel cellular immunotherapy. Various clinical trials reported severe side effects, such as neurotoxicity, on-target off-tumor activity, tumor lysis syndrome, and cytokine release syndrome (CRS), some of which were proportionally intense to the patients’ tumor burden [8–10]. At present, manufacturing of CAR T cells is commonly based on engineering patient-derived autologous T cells by integrating a CAR via retroviral or lentiviral gene transfer [11, 12] under good manufacturing practice (GMP) [13]. While retroviral vectors have been linked with insertional oncogenesis in the past [14], this phenomenon has not been observed in the context of CAR T cell therapy. Nonetheless, targeted CAR integration, e.g. by use of vectors based on adeno-associated viruses (AAV) in combination with designer nucleases, might provide a safer solution with beneficial CAR expression profiles [15, 16]. The use of targeted genome editing has yielded promising approaches with knockouts of *TRAC* [15, 17, 18], *CD52* [18, 19], and/or PD-1 [20], to generate CAR T cells with additional features, such as absent allo-reactivity, resistance to the clinically used CD52 antibody alemtuzumab or higher persistence, respectively. Designer nuclease technology opens up further opportunities in the CAR T cell space, including the integration of genetic or immunologic safety switches, such as iCasp or RQR8, respectively, that allow for positive or negative selection of the engineered cells during manufacturing or post-infusion in the patient. RQR8 in particular is an immunological safety switch composed of QBEnd10 [21], a CD34 epitope that can be used for positive selection during manufacturing, two CD20 mimotopes that can be targeted with the FDA-approved CD20 antibody rituximab [22–25], and a CD8 stalk for membrane anchoring (Fig. 1a).

In this study, we aimed at targeted epitope switching to produce CAR T cells that can be positively and/or negatively selected during both the manufacturing process, and that have the potential for antibody-based selection upon infusion in the patient. To this end, we combined CRISPR-Cas9 technology with an AAV-based vector system [26] to mediate targeted integration of a CAR–RQR8 expression cassette into the *CD52* locus of primary T cells (Fig. 1a). We demonstrate that the *CD52* locus enables highly efficient genome editing and that the resulting epitope-switched CAR (esCAR) T cells, CD20^−^/CD34^−^/CD52^+^ → CD20^+^/CD34^+^/CD52^−^, can be used to simplify enrichment of gene-edited cells during manufacturing by employing magnetic bead-based selection strategies. Furthermore, we show that the esCAR T cells are resistant to the T lymphocyte-depleting alemtuzumab whilst being susceptible to the B cell-depleting antibody rituximab. Hence, while retaining potent killing capacities against CD19^+^ tumor cells in combination with secretion of expected cytokines upon antigen sensitization, these esCAR T cells harbor valuable selection features for simplified manufacturing protocols and the potential for specific in vivo depletion.

## Materials and methods

### Antibodies

The following antibodies were used: anti-human CD52-FITC (clone HI186, BioLegend, San Diego, California, USA, cat# 316004), CD19-CAR Detection Reagent (Miltenyi Biotec, Bergisch Gladbach, Germany, cat# 130-115-965), anti-Biotin-PE (clone REA746, Miltenyi Biotec, cat# 130-110-951), anti-Biotin-FITC (Miltenyi Biotec, cat# 130-090-857), anti-human CD34 (QBEnd10-APC, R&D Systems, Minneapolis, Minnesota, USA, cat# FAB7227A), anti-human CD25-PE (clone 4E3, Miltenyi Biotec, cat #130-113-282), alemtuzumab (Genzyme Corporation, Cambridge, Massachusetts, USA, NDC 58468-0357-3), rituximab (Roche, Basel, Switzerland, PZN #8709904), anti-human IgG goat F(ab′)2-PE (SouthernBiotech, Birmingham, Alabama, USA, cat# 204209). For live/dead staining 7-aminoactinomycin D (7-AAD, Invitrogen, Carlsbad, California, USA, cat# A1310) and propidium iodide (PI, Sigma-Aldrich, St. Louis, Missouri, USA, cat# P4170) were used. DPBS (Thermo Fisher Scientific/GIBCO, Waltham, Massachusetts, USA, cat# 14190250) was used for washing and for FACS buffer preparation (DPBS, 5% FBS, 0.1% Na^+^-Azide and 2 mM EDTA).

### AAV vector production

The HDR donor was cloned into plasmid pSUB201 [27] using NEBuilder HiFi DNA Assembly Master Mix (NEB, Ipswich, Massachusetts, USA, #E2621S). It consists of a homology arm (HA) left (482 bp) and a HA right (488 bp) to *CD52* (both of which were amplified from genomic DNA of primary T cells), CD19-CAR (FMC63) [28], P2A [29], and RQR8 [24]. AAV6 vector particles were produced as described previously [30], and AAV vector titers assessed by ddPCR [31].

### Cells and media

All cells were cultured at 37 °C in the presence of 5% CO_2_. Peripheral blood mononuclear cells (PBMCs) were isolated from leukocyte reduction system chambers by density gradient centrifugation (Ficoll-Paque). PBMCs and primary T cells were cultured in RPMI 1640 medium (Thermo Fisher Scientific/GIBCO, cat# 61870-010) supplemented with 10% fetal calf serum (FBS, PAN-Biotech, Aidenbach, Germany, cat# P40-47500), 1% penicillin-streptomycin (P/S, Sigma-Aldrich, cat# P0781), 10 mM HEPES (Sigma-Aldrich, cat# H3375), and human interleukin-2 (IL-2) 100 U/ml (ImmunoTools, Friesoythe, Germany, cat# 11340027). Human IL-7 (Miltenyi Biotec, cat# 130-095-361) at 25 U/ml and IL-15 (Miltenyi Biotec, cat# 130-095-762) at 50 U/ml were added to the media to culture the edited T cells. HEK293T cells (ATCC, Manassas, Virginia, USA, CRL-3216™) were cultured in DMEM high-glucose GlutaMAX medium (Thermo Fisher Scientific/GIBCO, cat# 10566016) supplemented with 10% FBS, 1% P/S, and 1% sodium pyruvate (Biochrom, Cambridge, United Kingdom, cat# L0473). NALM6 (DSMZ, Braunschweig, Germany, cat# ACC128) and K562 (DSMZ, cat# ACC10) cells were cultured in RPMI 1640 medium supplemented with 10% FBS and 1% P/S. The cell lines were expanded from thawed vials of low-passage cells, showed expected morphology and underwent monthly mycoplasma screens which all turned out negative. STR profiling was not regularly performed.

### Genetic engineering of T cells

PBMCs were thawed and activated using ImmunoCult Human CD3/CD28/CD2 T-cell Activator (5 µl/10^6^ cells) (STEMCELL Technologies, Vancouver, Canada, cat# 10970). Three days later, ribonucleoprotein (RNP) complexes were transferred to 1 × 10^6^ cells using the EO-115 program (4D-Nucleofector, Lonza, Basel, Switzerland) and kit P3 (Lonza, cat# V4XP-3032) following the manufacturer’s instructions. Prior to electroporation, 20 pmol of SpCas9 protein (PNA Bio, Newbury Park, California, USA, cat# CP02) and 112.5 pmol of synthetic gRNA (Synthego, Silicon Valley, California, USA) were complexed by incubation for 10 min at RT. Upon electroporation, fresh complete medium with 1000 U/ml of IL-2 was added to recover the cells. The cells were split in four wells (0.25 × 10^6^ cells/well) of a 96-well U-shaped-bottom microplate (Falcon, Fisher Scientific, Hampton, New Hampshire, USA, cat# 08-772-54) and transduced with indicated amounts of AAV vector particles 15 min post electroporation.

### Genotyping

T7 endonuclease 1 (T7E1) assays were performed as previously described [32]. In short, genomic DNA was extracted using the QIAamp DNA Blood Mini Kit (Qiagen, Hilden, Germany, cat# 51104). The *CD52* target region was amplified by PCR on 100 ng of genomic DNA using primers CD52(fwd) 5′-AGTCCCCTGATCTTATCCCA and CD52(rev) 5′-GGCACTGCCTGTCAACTTCT. For NGS, the genomic *CD52* target region was amplified as described above. NEBNext Ultra II DNA Library Prep Kit for Illumina (NEB, cat# E7645L) and NEBNext Multiplex Oligos for Illumina (NEB) were used for library preparation. A ddPCR Library Quantification Kit for Illumina TruSeq (AbD serotec Bio-Rad, Hercules, California, USA, cat# 186-3040) was used to measure library concentrations. Subsequently, 4 pmol of the library was sequenced using the Illumina Miseq (Illumina, San Diego, California, USA), and data analyzed using the command line version of CRISPResso2 [33].

Targeted transgene integration was confirmed by in–out PCR using pairs of primers that cover either the 5′ or the 3′ junction sites of *CD52* (Fig. 1a): 5′end(fwd) 5′-TGTCAAAGCAGGAAGCAGTC; 5′end(rev) 5′-GCACAGCAGGCTGGTCAGCATAGGACCGGGGTTTTCCTC; 3′end(fwd) 5′-AACTTCTCGCTGCTGAAGCAGG; 3′end(rev) 5′-ATGTCCAGGGTGGTGGCATC. PCR reactions included 10 μM dNTP mix, 10 μM of each primer, 5X Phire Reaction Buffer, and Phire Hot Start II DNA polymerase (Thermo Fisher Scientific, cat# F122S) with 100 ng of genomic DNA in 25 µl. PCR conditions were as follows: 98 °C for 5 min, 30 cycles at 98 °C for 30 s, 66 °C for 30 s, 72 °C for 72 s (5′)/42 s (3′), and a final elongation at 72 °C for 5 min.

### Flow cytometric analysis

For staining, 1 × 10^5^ cells were washed and stained with the specified antibodies in FACS buffer at 4 °C in the dark for 20–30 min. Whenever staining included CD19-CAR Detection Reagent, incubation was carried out at RT in the dark for 40 min. A BD Accuri C6 flow cytometer (BD Biosciences, San Jose, California, USA) was used for data acquisition; FlowJo V10 (BD Biosciences) was used for data analysis.

### Complement-dependent cytotoxicity (CDC) assay

T cells were plated at 1 × 10^5^ cells/well (96 flat-bottomed well, Sarstedt, Nümbrecht, Germany, cat# 83.3924.500) in 200 µl of complete RPMI 1640 media, in the presence or absence of antibody (alemtuzumab,10 µg/ml; rituximab 50 µg/ml) and/or 20 µl of baby rabbit complement (AbD serotec Bio-Rad, cat# C12CA). Cells were incubated for 2 h at 37 °C and then stained with 7-AAD to determine cytotoxicity by flow cytometry on a BD Accuri C6 flow cytometer.

### CAR T cell-mediated cytotoxicity and cytokine release

Overall, 1 × 10^4^ effector esCAR T cells and 1 × 10^4^ target cells (NALM6-GFP, K562-GFP) were co-cultured in complete medium without cytokines in 96-well U-shaped-bottom microplate in RPMI 1640 media at effector-to-target (E:T) ratios of 1:1. After 1 and 2 days, cells were collected, PI was added to exclude dead cells, and the numbers of live GFP^+^ cells assessed by flow cytometry on a BD Accuri C6 flow cytometer. The supernatants of these cells were collected at indicated time points and assayed by cytometric bead array (CBA, BD Biosciences) to determine the extent of IFN-γ, GM-CSF, and granzyme B release according to the manufacturer’s instructions. A BD FACSCanto II flow cytometer (BD Biosciences, San Jose, California, USA) was used for data acquisition; FlowJo V10 was used for data analysis.

### Magnetic bead selection of esCAR T cells

esCAR T cells were purified using CD34 MicroBead kit UltraPure human (Miltenyi Biotec, cat# 130-100-453) with the MACS-LD column (Miltenyi Biotec, cat# 130-042-901) according to the manufacturer’s instructions. The extent of enrichment was checked by flow cytometry on a BD Accuri C6 flow cytometer.

### Statistics

All experiments shown were replicated at least three times as independent experiments, except for the experiments in Figs. 4c–f and S3c–e, which represent data of three technical replicates of single experiments (*N* = 1). Unless indicated otherwise, ANOVA tests (two-way, equal variance) were performed to determine statistically significant differences using Tukey’s multiple comparison. For statistical analysis in Fig. 3e a Student’s *t* test (unpaired, equal variance, two-tailed) was used. *, **, and *** indicate *P* < 0.05, *P* < 0.01, *P* < 0.001, respectively. The center values reported are the mean and error bars represent standard deviation of at least three independent experiments, if not indicated otherwise. All data meet the assumptions of the tests used. No data were excluded from analysis due to outlying data points. GraphPad 8.4.3 (GraphPad, San Diego, California, USA) was used for data analysis and creation of graphs.

## Results

### Efficient gene targeting in primary T cells

In order to target *CD52*, several gRNAs were designed to direct Cas9 nuclease to exon 2 of the locus (data not shown). RNP complexes of the best-performing CRISPR-Cas nuclease were transferred to T cells derived from primary PBMCs using electroporation. Genotyping by T7E1 assay confirmed efficient formation of insertion/deletion (indel) mutations at the target site (Fig. 1b), which was validated by next-generation targeted amplicon sequencing (Fig. 1c). More than 80% of alleles contained mutations, with +1 and −2 indels being the most frequent alleles (Fig. S1a), which might explain the discrepancy between T7E1 and NGS data. In parallel, a second-generation CD19-targeting CAR (FMC63), which was linked to RQR8 using a P2A sequence, was cloned into an AAV backbone for packaging into an AAV serotype 6 (AAV6) vector. This AAV6 donor harbored some 500 bp long HAs to the *CD52* target region. The CAR-P2A-RQR8 cassette is under the control of a short elongation factor 1α promoter, which ensured stable expression of the construct (Fig. 1a). To target integration of CAR-P2A-RQR8 into *CD52*, 1 × 10^6^ activated T cells were electroporated with RNPs, immediately followed by transduction with increasing amounts of AAV6 particles (1 × 10^4^ to 5 × 10^4^ vector copies per cell). Edited T cells were cultured for 22 days and analyzed by genotyping. As assessed by T7E1 assay, the frequency of edited *CD52* target sites was comparable across all samples (≈45% of indels), independent of the amount of AAV (Fig. 1b). 5′ and 3′ junction in–out PCR reactions were performed to confirm targeted integration of the CAR-P2A-RQR8 construct into the *CD52* locus (Fig. 1d). The PCR amplicons were extracted from the gel and sequence analysis confirmed correct integration of the construct (data not shown).

**Fig. 2:**
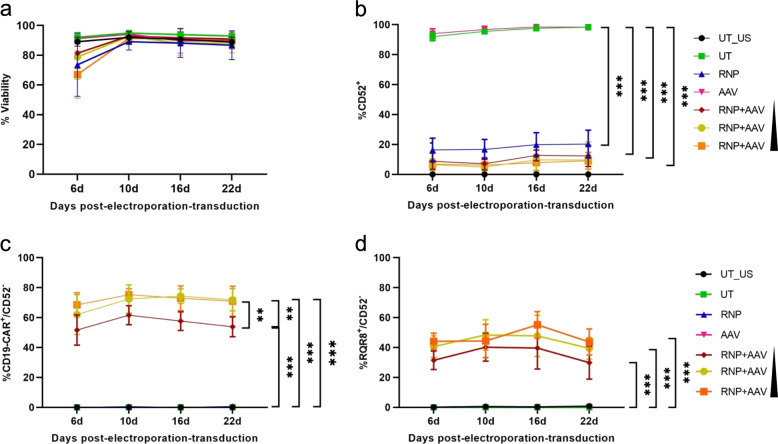
Genome editing in primary T cells — phenotypic analysis. **a** Viability. Viabilities of treated cells are shown for different time points. **b** CD52 expression. The percentages of CD52^+^ cells were determined by flow cytometry at different time points. **c** CAR expression. The percentages of CAR^+^/CD52^−^ T cells were determined at indicated time points. **d** RQR8 expression. The percentages of RQR8^+^/CD52^−^ T cells were determined at indicated time points. UT_US, untreated T cells unstained; UT, untreated T cells; RNP, RNP-electroporated T cells; AAV, AAV-transduced T cells RNP + AAV, RNP-electroporated T cells transduced with 1 × 10^4^, 3 × 10^4^, and 5 × 10^4^ genome copies/cell. ** and *** indicate *P* < 0.01 and *P* < 0.001, respectively. The center values reported are the mean and error bars represent standard deviation (SD) of at least three independent experiments (*N* > 3).

Phenotypic analyses of the gene-edited T cells by flow cytometry did not reveal any substantial toxicity of our treatment conditions, even with the highest amount of AAV6 particles (Fig. 2a). Viability increased from >60% at day 6 post-treatment to 80–90% from day 10 on. Assessment of CD52 expression confirmed effective and stable genome editing with up to 90% of T cells being CD52^–^ for at least 22 days (Fig. 2b). Moreover, the CD19-CAR/RQR8 transgene was stably expressed, with up to 75% of T cells being CAR^+^/CD52^−^ (Fig. 2c) and up to 60% of lymphocytes RQR8^+^/CD52^−^ (Fig. 2d) for at least 22 days. CAR/RQR8 double staining proved that almost all RQR8^+^ T cells expressed the CD19-CAR (Fig. S1b). Together, the experiments confirm that a single production step could generate up to 80% of esCAR T cells without further selection.

### esCAR T cells are alemtuzumab resistant and rituximab sensitive

**Fig. 3:**
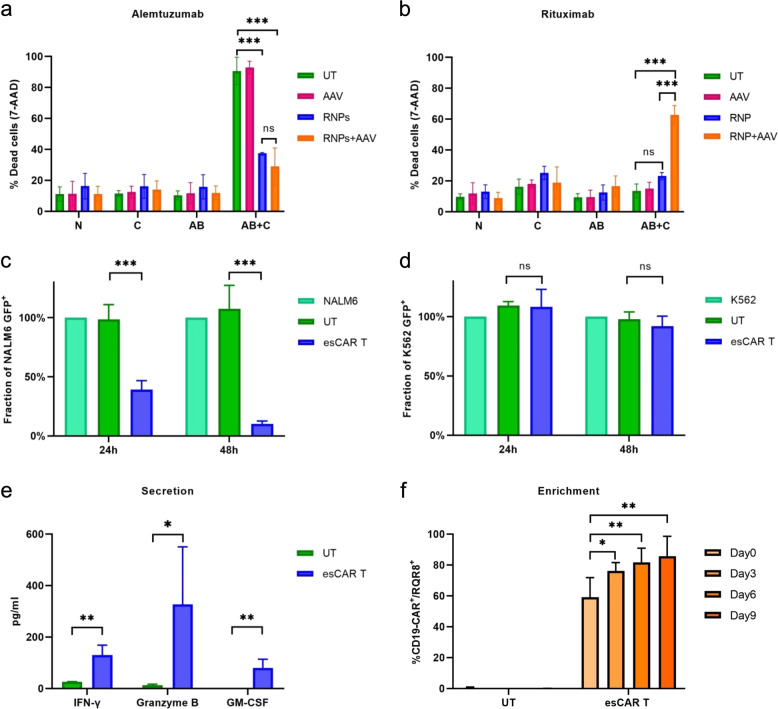
Functional characterization of esCAR T cells. **a**, **b** Sensitivity to alemtuzumab or rituximab. Complement-dependent cytotoxicity (CDC) assay was performed with media only (N), complement only (C), antibody only (AB), or antibody plus complement (AB + C). Dead cells were identified by 7-AAD staining. UT, untreated T cells; AAV, AAV-transduced T cells; RNP, RNP-electroporated T cells; RNP + AAV, electroporated with RNP and transduced with 5 × 10^4^ genome copies/cell. **c**, **d** Cytotoxicity assays. Effector esCAR T cells or untreated T cells (UT) were co-cultured with NALM6-GFP (CD19^+^) or K562-GFP (CD19^–^) target cells at effector-to-target (E:T) ratios of 1:1. Percentages of viable GFP^+^ cells are shown for 24 and 48 h time points. Fraction of GFP^+^ cells was normalized to the target-only sample (NALM6, K562). **e** Secreted factors. Supernatants of cytotoxicity assays (**c**) at 24 h of co-culture were used to assess the release of IFN-γ, granzyme B, or GM-CSF by cytometric bead array. **f** Enrichment of RQR8-positive cells. esCAR T cells or untreated T cells (UT) were co-cultured with irradiated NALM6 cells for up to 9 days. The percentages of RQR8^+^/CAR^+^ T cells were determined at indicated time points. *, **, and *** indicate *P* < 0.05, *P* < 0.01, *P* < 0.001, respectively. The center values reported are the mean and error bars represent standard deviation (SD) of at least three biological independent experiments (*N* > 3).

The engineered esCAR T-cell phenotype (CD52^−^/RQR8^+^) should give rise to CD19-targeting T cells that are simultaneously alemtuzumab-resistant and rituximab-sensitive. CDC assays were performed to confirm those features. In the presence of complement, either antibody mediated killing of cells that expressed the corresponding epitope (Fig. 3a, b). As determined by 7-AAD staining, alemtuzumab induced cell death in 90–95% of the T cell population for both untreated (UT) cells and the samples transduced with AAV6 donor in the absence of RNP (AAV). In contrast, CRISPR-Cas-mediated *CD52* knockout (RNP) or targeted integration of the CAR–RQR8 cassette (RNP + AAV) provided resistance to some 60%–70% of T cells, respectively (Figs. 3a and S2a). No significant cell killing was observed with either “media only” (N), “complement only” (C), or “antibody only” (AB). To verify that cell killing was specific to CD52^+^ T cells, flow cytometry was performed prior to (pre-CDC) and after (post-CDC) treatment (Fig. S2b). The experiment confirmed that CD52^+^ but not CD52^–^ cells were sensitive to alemtuzumab mediated CDC, allowing enrichment of the edited T-cell population to almost 100%.

Using CD20-targeting rituximab in the CDC assay, we induced cell death of esCAR T cells expressing RQR8 (Fig. 3b). No dead cells could be detected in the control samples. Again, flow cytometric analyses of pre- and post-rituximab-CDC samples confirmed specific elimination of RQR8^+^ T cells (Fig. S2c). In conclusion, we could show that esCAR T cells are resistant to alemtuzumab and sensitive to rituximab in vitro.

### esCAR T cells effectively kill CD19^+^ tumor cells

To prove that the engineered esCAR T cells remain functional, we co-cultured esCAR T cells with CD19^+^ NALM6 cells and CD19^–^ K562 cells. In order to facilitate the readout of the cytotoxicity assays, these cell lines constitutively express GFP, so allowing us to quantify CD19-targeted cell killing by tracing the depletion of GFP^+^ cells over time. Control T cells (UT) and esCAR T cells were co-cultured with either NALM6-GFP or K562-GFP cells at a 1:1 E:T ratio, and targeted killing was assessed after 24 and 48 h, respectively. At both time points, supernatants were collected for analyses of IFN-γ, GM-CSF, and granzyme B secretion. esCAR T cells effectively eliminated NALM6 cells, reaching ≈90% depletion of the GFP^+^ cells after 48 h (Fig. 3c). In contrast, when esCAR T cells were co-cultured with K562 cells, the numbers of GFP^+^ cells remained unchanged (Fig. 3d). Analyses of the supernatants revealed release of IFNγ, granzyme B, and GM-CSF from esCAR T cells but not UT control cells (Fig. 3e).

### Selected and enriched esCAR T cells maintain their features and function

Of note, the fraction of CD19-CAR and RQR8-positive esCAR T cells seemed to diverge by 10–20% (Figs. 2c, d and S1b), which was in line with results we obtained from γ-retroviral transduction experiments unrelated to this study. In that case, retroviral delivery of an RQR8/dLNGFR construct revealed an even higher disagreement in expression levels between the two proteins (Fig. S3a). We hence hypothesized that this discrepancy is mainly attributed to divergent antibody staining efficiencies but we could not rule out that the T cell activation status might contribute to the observed differences. We therefore challenged esCAR T cells by continuous antigen exposure through sequential co-culture with irradiated NALM6 cells for up to 9 days. At different time points, we traced esCAR T-cell expression levels of CD25, CD19-CAR, and RQR8. CD25 staining revealed a strong activation of esCAR T cells (Fig. S3b), which seemed to correlate with enhanced RQR8 expression levels, with >80% of the expanded esCAR T cells staining double positive for CD19-CAR and RQR8 (Fig. 3f). Importantly, continuous challenge of esCAR T cells by sequential co-culture with CD19^+^ target cells did neither affect resistance to alemtuzumab nor susceptibility to rituximab (Fig. S3c, d).

**Fig. 4:**
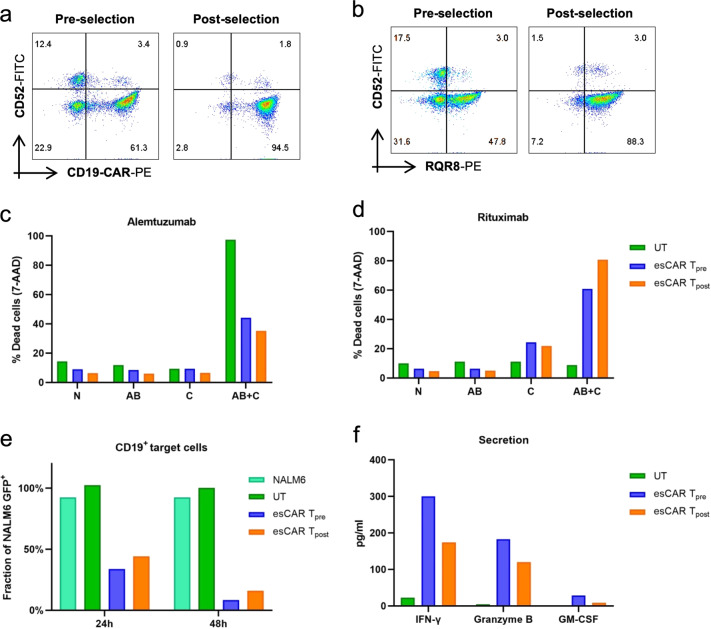
Functional characterization of positively selected esCAR T cells. esCAR T cells were enriched by magnetic bead selection using QBEnd10. **a**, **b** Visualization of enrichment. Shown are representative flow cytometric analyses. **c**, **d** Sensitivity to alemtuzumab or rituximab. Complement-dependent cytotoxicity (CDC) assays were performed with media only (N), complement only (C), antibody only (AB), or antibody plus complement (AB + C). Dead cells were identified by 7-AAD staining. **e** Cytotoxicity assay. Effector esCAR T cells or untreated T cells (UT) were co-cultured with NALM6-GFP (CD19^+^) target cells at effector-to-target (E:T) ratios of 1:1. Percentages of GFP^+^ cells are shown for 24 and 48 h time points. The fraction of GFP^+^ cells was normalized to the target-only sample (NALM6). **f** Secreted factors. Supernatants of cytotoxicity assay (**e**) at 24 h of co-culture were used to assess the release of IFN-γ, granzyme B, or GM-CSF by cytometric bead array. UT, untreated T cells; esCAR T_pre_, esCAR T cells preselection; esCAR T_post_, esCAR T cells post-selection. The center values reported are the mean of three technical replicates (*N* = 1).

The epitope spectrum of esCAR T cells provided by RQR8 facilitates enrichment of gene-edited T cells using commercially available selection systems. Using a QBEnd10-based system to select for CD34^+^ cells yielded an almost 90% pure CD19-CAR^+^/RQR8^+^ esCAR T cell population (Fig. 4a, b). To validate that selection did neither affect the desired sensitivity nor resistance features nor function of esCAR T cells, CDC assays and cytotoxicity assays were performed to compare esCAR T cells prior to and after selection. The CDC assays proved that esCAR T cells exhibited similar levels of resistance to alemtuzumab or susceptibility to rituximab post-selection as their corresponding counterparts preselection (Fig. 4c, d). As expected, however, post-selection esCAR T cells were slightly less sensitive to alemtuzumab than preselection, reflecting the reduced fraction of CD52^+^ cells (Fig. 4c). Inversely, the higher frequency of dead cells in post-selection esCAR T cells mirrored the higher fraction of rituximab-sensitive RQR8^+^ esCAR T cells (Fig. 4d). No significant differences were observed in the cytotoxic activity of preselected versus post-selected esCAR T cells. Both populations elicited high effector activity against CD19^+^ tumor cells (Fig. 4e) but not CD19^–^ cells (Fig. S3e), underlining that CD34 epitope-based selection preserved functionality of esCAR T cells. Effector activity was confirmed by measuring cytokine release. Both esCAR T_pre_ and esCAR T_post_ cells released similar amounts of IFNγ, granzyme B, and GM-CSF when exposed to CD19^+^ tumor cells (Fig. 4f). Hence, QBEnd10-based magnetic bead selection represents a simple and effective method for the purification of esCAR T-cell populations that does neither interfere with their function nor with their immunogenic features.

## Discussion

Although CAR T cell therapy has been successfully introduced to the clinic for a variety of hematologic malignancies [4, 5], it is still linked to immunological side effects that affect the overall outcome of the patients [9, 10]. For instance, CRS is a common and severe immunological side effect that is based on the release of high concentrations of cytokines by activated CAR T cells. Many efforts have been made to engineer CAR T cells to mitigate or control these symptoms. In this study, we genetically edited CD19-targeting CAR T cells to introduce an immunological safety switch that enables positive or negative selection both in vitro during manufacturing and in vivo after administration to the patient. While some groups demonstrated that the disruption of *CD52* conferred resistance to alemtuzumab [18, 19], other labs have proven RQR8 to be useful for the selective elimination of CAR T cells by rituximab both in vitro and in vivo [18, 24]. Here, we demonstrate for the first time that CD52 can be replaced with the multi-epitope safety switch RQR8 in a single step with high efficacy by combining CRISPR-Cas9 with AAV vector technology. We established that the resulting esCAR T cells maintain their ability to kill CD19^+^ target cells and that replacing CD52 with RQR8 rendered esCAR T cells susceptible to rituximab and resistant to alemtuzumab.

CD52 is present on the surface of mature lymphocytes. Binding of the CD52 antibody alemtuzumab to lymphocytes was shown to prompt their elimination via complement-mediated cytotoxicity [34]. In clinical practice, alemtuzumab is used, e.g., to induce lymphodepletion in recipients of hematopoietic stem cell transplantation to prevent graft rejection [35, 36], or as a treatment for B-cell chronic lymphocytic leukemia (B-CLL) [37]. On the other hand, the chimeric monoclonal CD20 antibody rituximab is used to treat certain autoimmune disorders and B cell-based malignancies, such as non-Hodgkin lymphoma and B-CLL, by inducing the depletion of B lymphocytes [38, 39]. Replacing CD52 with RQR8 in CD19-targeting CAR T cells would hence protect the esCAR T cells during ongoing alemtuzumab therapy, as, e.g., indicated after stem cell transplantation. On the other hand, the sensitivity toward rituximab provides a useful safety feature. If signs of CAR T cell-based severe side effects arise, the engineered T lymphocytes could be eliminated effectively by infusion of rituximab. A previous clinical study has faced problems eliminating all CAR T cells infused to the patients when using CD19-CAR T cells that co-expressed an RQR8 safety switch, suggesting system “leakiness” [18]. Other classes of suicide switches have, too, shown such evasion of intended full CAR T cell depletion [40]. Further studies will have to elucidate whether the targeted integration principle of esCAR T cells performs better and yields higher depletion efficiencies in vivo. In this in vitro study, almost all RQR8^+^ T cells could be removed upon rituximab treatment. We attribute this observation to the design of our CAR-P2A-RQR8 cassette, which allows us to enrich for QBEnd10, yielding a highly enriched fraction of rituximab-sensitive RQR8^+^ cells. We hypothesize that a close-to-complete depletion of CAR T cells would significantly alleviate potential side effects. Of note, rituximab-induced B-cell depletion does not affect memory B and plasma cells, so ensuring recovery of the B-cell lineage after discontinuation of antibody treatment [38, 41].

Although the expression of CAR and RQR8 is genetically linked, we observed differences between CD19-CAR and RQR8 expression levels on the cell surface. Using a similar construct, others have shown before that RQR8 expression was lower than a genetically linked CAR expression [18]. While we cannot exclude that the used antibodies bind these markers with diverse affinity/avidity, we speculate that RQR8 is not transported well to the cell surface or unstable. As shown here, activation of esCAR T cells by exposure to antigen-bearing cells seems to augment both RQR8 expression levels as well as the fraction of RQR8^+^ cells, suggesting that T cell activation has a beneficial effect on RQR8 transport or stability.

AAV vectors constitute a particularly good template for genome editing that aims at targeting CAR integration to particular loci in primary T cells [15, 17, 42]. Semi-random integration of transgenes, as with retroviral or lentiviral vectors, can potentially generate undesired outcomes with unpredictable genotypes and abnormal phenotypes [14]. To our knowledge, designer nuclease-mediated targeted integration with AAV vectors has not been linked with insertional oncogenesis, yet still enables highly efficient gene editing. In this study, we reached gene targeting frequencies of up to 70% with minimal cytotoxicity and prior to selection. Moreover, CAR transgene expression was stable for at least 1 month. Novel CAR T cell manufacturing strategies, namely CD4/CD8 preselection from leukapheresis products, facilitated CAR T cell production by yielding higher transduction efficiencies and increasing CAR T cell product manufacturing feasibility [43]. Future studies will have to investigate the transferability of this principle to our esCAR T cell strategy. Further developments may include the combination of targeting the integration of the CD19-CAR/RQR8 construct with the well-described concept of “universal” CAR T cells, i.e., creating allo-transplantable CAR T products by integration into the *TRAC* locus.

High gene targeting frequencies in primary T lymphocytes were reported before for *TRAC* and *CCR5* [15, 17, 42], suggesting that the high efficacies we observed here are not related to the *CD52* locus but to a cellular environment that supports HDR. On the other hand, our data suggest random integration of the CAR–RQR8 cassette, albeit at low frequencies (1–3%). This is in agreement with previous reports from us and others that demonstrated that AAV vectors can integrate into DNA double strand breaks using the nonhomologous end-joining pathway [44–47]. We do not believe that this fact will affect the overall functionality and safety of esCAR T cells in vivo because rituximab treatment would also deplete CD52^+^ CAR T cells.

In conclusion, the established one-step strategy to generate CD19-targeting esCAR T cells that are resistant to alemtuzumab and sensitive to rituximab can be easily implemented in “GMP” protocols for prospective applications in patients bearing CD19^+^ cancer cells.

## Supplementary information

Supplementary Material
