# Supplementary material for: Targeted multi-epitope switching enables straightforward positive/negative selection of CAR T cells
Source: Gene Ther. 2021 Feb 1;28(9):602–12. doi: 10.1038/s41434-021-00220-6 (PMC8455323; doi:10.1038/s41434-021-00220-6)
Supplement: Supplementary file 1 — Supplementary Material [file 41434_2021_220_MOESM1_ESM.pdf]

SUPPLEMENTARY DATA

Targeted multi-epitope switching enables straightforward positive/negative selection of CAR T cells

Laura Mosti[1,2,3], Lukas M. Langner[1,2,3], Kay O. Chmielewski[1,2,3], Patrick Arbuthnot[4], Jamal Alzubi[1,2,3] and Toni Cathomen[1,2,3,#]

[1] Institute for Transfusion Medicine and Gene Therapy, Medical Center - University of Freiburg, Freiburg, Germany.

[2] Center for Chronic Immunodeficiency (CCI), Medical Center - University of Freiburg, Freiburg, Germany

[3] Faculty of Medicine, University of Freiburg, Freiburg, Germany

[4] Wits/SAMRC Antiviral Gene Therapy Research Unit, School of Pathology, Faculty of Health Sciences, University of the Witwatersrand, Johannesburg, South Africa

[#] Correspondence: Toni Cathomen, toni.cathomen@uniklinik-freiburg.de

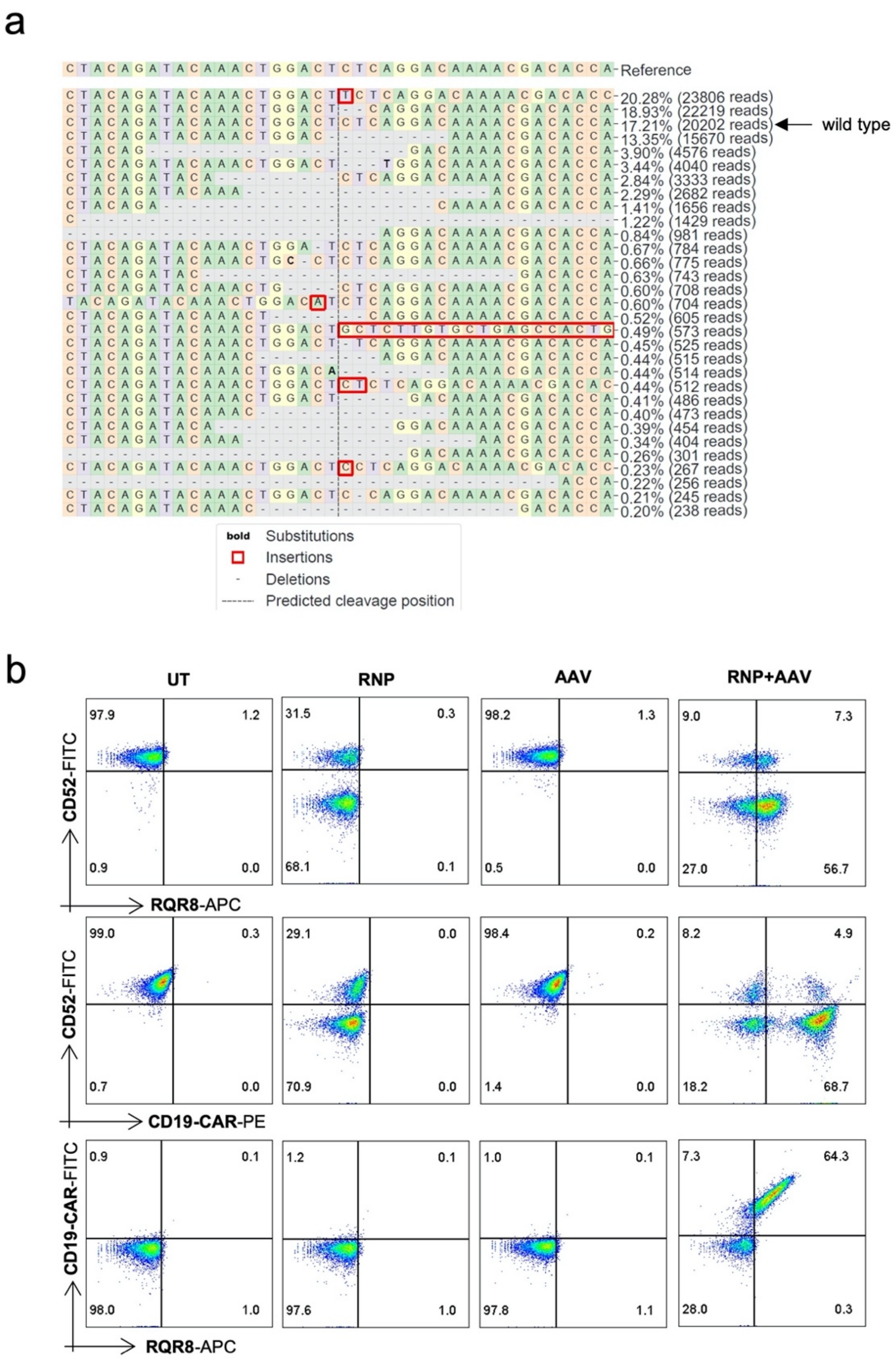

Figure S1: Profile of esCAR T cells. a Genotypic analysis by NGS. Data were analyzed using CRISPResso2. Exemplarily shown is the analysis of the RNP-electroporated sample. The reference target sequence of *CD52* is indicated on top, followed by the different modified and unmodified sequences ranked according to their number of reads. The arrow pinpoints the wild type sequence. **b** Phenotypic analysis by flow cytometry. Exemplarily shown are double stainings that reveal the expression of CD52, RQR8 and CD19-targeting CAR. UT, untreated T cells; RNP, RNP-electroporated T cells; AAV, AAV-transduced T cells; RNP+AAV, T cells electroporated with RNP and transduced with $5x10^4$ genome copies/cell.

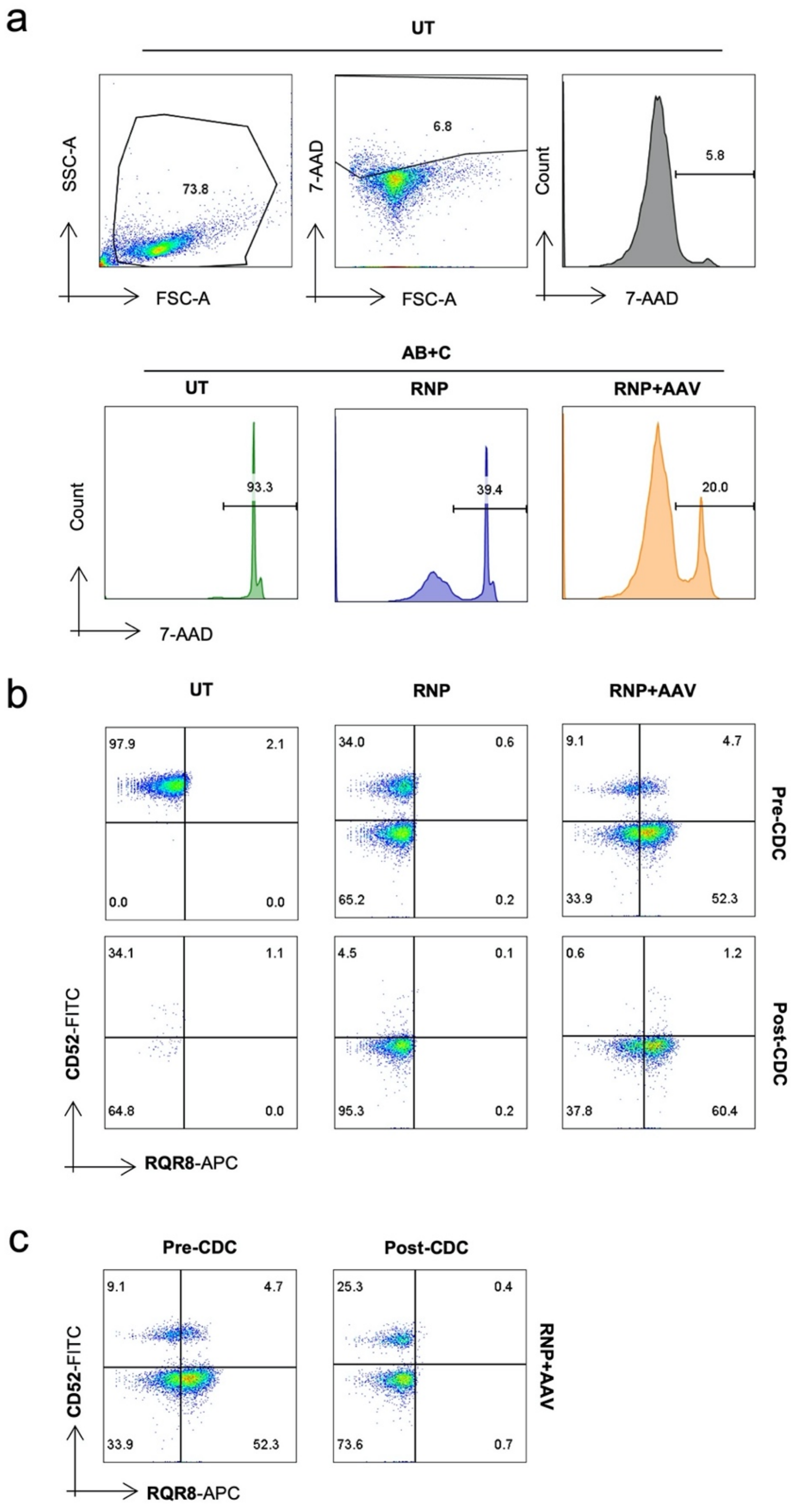

Figure S2: Complement-dependent cytotoxicity (CDC) assay. a Gating strategy. Cells were pre-gated by scatter plotting to exclude cell debris, followed by 7-AAD detection, which was also plotted as histograms. **b-c** Selective killing in CDC assay. Shown are double staining for CD52 and RQR8 before (pre-CDC) and after (post-CDC) addition of Alemtuzumab (b) or Rituximab (c). UT, untreated T cells; RNP, RNP only; AAV, vector only; RNP+AAV, electroporated with RNP and transduced with $5x10^4$ genome copies/cell.

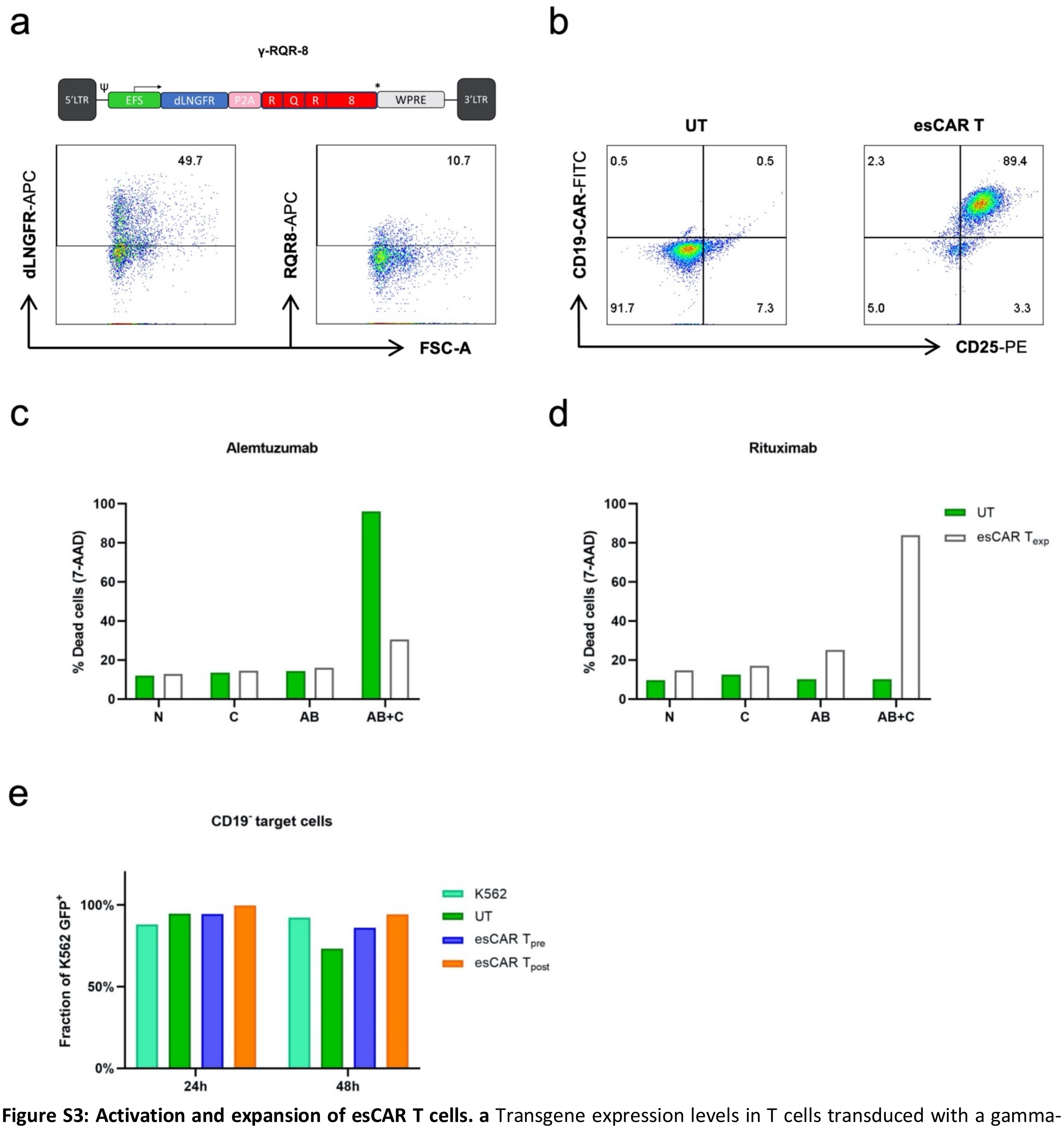

Figure S3: Activation and expansion of esCAR T cells. a Transgene expression levels in T cells transduced with a gamma-retroviral vector carrying a dLNGFR-P2A-RQR8 cassette. Flow cytometric analysis was performed 5 days post-transduction. **b** Activation of esCAR T cells. Untreated T cells (UT) or esCAR T cells were challenged by co-cultivation with irradiated NALM6 target cells. Expression of CD19-CAR and CD25 was assessed by flow cytometry after 6 days. **c-d** Sensitivity of expanded esCAR T cells to Alemtuzumab or Rituximab. Complement-dependent cytotoxicity assay was performed with media only (N), complement only (C), antibody only (AB), or antibody plus complement (AB+C). UT, untreated T cells; esCAR T_{exp}, expanded esCAR T cells. **e** Cytotoxicity assay. Effector esCAR T cells (pre- and post-selected) or untreated T cells (UT) were co-cultured with K562-GFP ($CD19^-$) target cells at effector to target (E:T) ratios of 1:1. Percentages of GFP^+ cells are showed at 24h and 48h time points. The fraction of GFP^+ cells was normalized to the target-only sample (K562). The center values reported are the mean of three technical replicates (N=1).
